# Methodological framework to identify possible adverse drug reactions using population-based administrative data

**DOI:** 10.12688/f1000research.4816.1

**Published:** 2014-10-29

**Authors:** Brian Sauer, Jonathan Nebeker, Shuying Shen, Randall Rupper, Suzanne West, Judith A. Shinogle, Wu Xu, Kathleen N. Lohr, Matthew Samore

**Affiliations:** 1 Informatics, Decision Enhancement, and Surveillance (IDEAS) Center, VA Salt Lake City Health Care System, Salt Lake City, UT, UT 84148, USA; 2 RTI International, Research Triangle Park, NC 27709-2194, USA; 3 The Maryland Institute of Policy Analysis and Research, University of Maryland, Baltimore, MD, MD 21250, USA; 4 Office of Public Health Informatics, Utah Department of Health, Salt Lake City, UT, UT 84116, USA

**Keywords:** adverse drug reactions, adverse drug events, drug safety, patient safety, Medicaid, pharmacoepidemiology, post-marketing surveillance, propensity scores, acetylcholinesterase inhibitors

## Abstract

**Purpose**: We present a framework for detecting possible adverse drug reactions (ADRs) using the Utah Medicaid administrative data. We examined four classes of ADRs associated with treatment of dementia by acetylcholinesterase inhibitors (AChEIs): known reactions (gastrointestinal, psychological disturbances), potential reactions (respiratory disturbance), novel reactions (hepatic, hematological disturbances), and death.

**Methods:** Our cohort design linked drug utilization data to medical claims from Utah Medicaid recipients. We restricted the analysis to 50 years-old and older beneficiaries diagnosed with dementia-related diseases. We compared patients treated with AChEI to patients untreated with anti-dementia medication therapy. We attempted to remove confounding by establishing propensity-score-matched cohorts for each outcome investigated; we then evaluated the effects of drug treatment by conditional multivariable Cox-proportional-hazard regression. Acute and transient effects were evaluated by a crossover design using conditional logistic regression.

**Results:** Propensity-matched analysis of expected reactions revealed that AChEI treatment was associated with gastrointestinal episodes (Hazard Ratio [HR]: 2.02; 95%CI: 1.28-3.2), but not psychological episodes, respiratory disturbance, or death. Among the unexpected reactions, the risk of hematological episodes was higher (HR: 2.32; 95%CI: 1.47-3.6) in patients exposed to AChEI. AChEI exposure was not associated with an increase in hepatic episodes. We also noted a trend, identified in the case-crossover design, toward increase odds of experiencing acute hematological events during AChEI exposure (Odds Ratio: 3.0; 95% CI: 0.97 - 9.3).

**Conclusions: **We observed an expected association between AChEIs treatment and gastrointestinal disturbances and detected a signal of possible hematological ADR after treatment with AChEIs in this pilot study. Using this analytic framework may raise awareness of potential ADEs and generate hypotheses for future investigations. Early findings, or signal detection, are considered hypothesis generating since confirmatory studies must be designed to determine if the signal represents a true drug safety problem.

## Introduction

Despite its limitations, the Food and Drug Administration’s (FDA) Adverse Drug Event Reporting System (FAERS) has successfully identified rare and unexpected adverse events ^1– 3^. In many previous studies, administrative data sources have been used to estimate the extent of the problem or confirm safety signals identified from AERS ^4, 5^. However, fewer studies have demonstrated the potential of administrative data for first-line adverse drug reaction (ADR) surveillance ^6^. In this pilot study, we present a framework for directed discovery of possible ADRs using population-based administrative data sources, an approach intended to complement the FDA’s adverse reporting system. We describe our approach as directed because we target specific health outcomes of interest instead of simply mining the data for statistical associations.

We examined the associations between drug use and possible ADRs resulting from treatment of dementia with acetylcholinesterase inhibitors (AChEIs), namely, donepezil hydrochloride, rivastigmine tartrate, and galantamine hydrobromide. We measured associations for four classes of ADEs—established reactions (gastrointestinal and psychological disturbance), potential reactions based on drug pharmacology (respiratory disturbance), novel unexpected reactions (hepatic and hematological disturbance), and death. Hepatic and hematologic syndromes were evaluated because they are two examples of potentially fatal reactions that have been found in post-marketing surveillance of drug-induced disease ^7^.

## Methods

The directed discovery framework consists of clinical framing, data preparation, event detection, and hypothesis generating and testing. The first three components are described in the Methods; hypothesis generating and testing are explored in the Discussion.

### Clinical framing and data preparation

Clinical framing consisted of reviewing the medical literature and consulting clinical experts to define the treatment groups, inclusion criteria, drug courses, outcomes and covariates.

***Sources.*** Data consisted of pharmacy and medical claims and enrollment status from Utah Medicaid recipients in the fee-for-service program between 1/01/2003 and 12/31/2005. We linked Utah death-certificate data to Medicaid recipients by a deterministic method using a social security number. To protect patients’ privacy, all potentially traceable personal identifiers were removed. The University of Utah Institutional Review Board approved this study (IRB_00016984).

***Subjects.*** We studied Utah Medicaid recipients’ aged 50 and older with a dementia-type diagnosis ( Table 1). As Medicaid enrollment occurs on a monthly basis, we tracked membership enrollment and de-enrollment and censored the patients whose enrollment was terminated and not re-established within the study period. Because of the relatively high rate of sustained enrollment, approximately 99% of the cohort was enrolled for at least 80% of the months from their first until their last month of eligibility or until the study period ended. We did not limit inclusion to continuously enrolled recipients.

**Table 1.** Dementia codes and targeted outcomes codes from the Healthcare Cost and Utilization Project.

HCUP CCS codes	Description
**Dementia** **diagnoses**	
5.3.1	Senile dementia; uncomplicated
5.3.2	Arteriosclerotic dementia
5.3.5	Pre-senile dementia; uncomplicated
5.3.6	Senile dementia with delirium
5.3.7	Other senility and organic mental disorders
**Gastrointestinal** **outcomes**	
9.4.3	Gastritis and duodenitis
9.4.4	Other disorders of stomach and duodenum
9.11	Non-infectious gastroenteritis
9.12.3	Other and unspecified gastrointestinal disorders
17.1.6	Nausea and vomiting
17.1.7	Abdominal pain
**Hematological** **outcomes**	
4.1	Anemia
4.2	Coagulation and hemorrhagic disorders
4.3	Diseases of white blood cells
4.4	Other hematological conditions
**Hepatic outcomes**	
9.8	Liver disease
**Psychological** **outcomes**	
5.4	Affective disorders
5.6	Other psychoses
5.7	Anxiety, somatoform, dissociative, and personality disorders
5.9	Other mental conditions
**Respiratory** **outcomes**	
8.2	Chronic obstructive pulmonary disease and bronchiectasis

**Figure 1.**
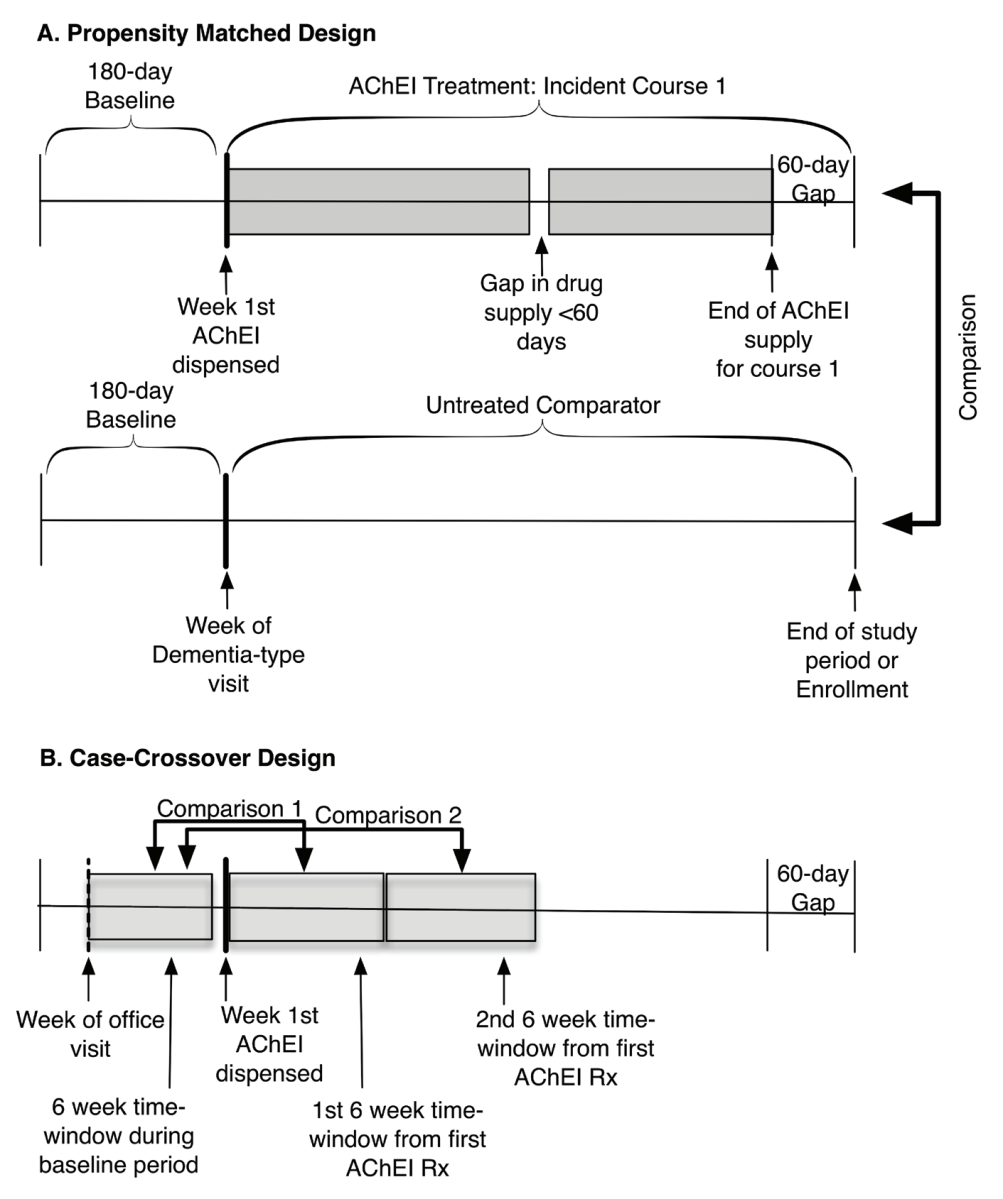
Treatment time-windows for cohort and case-crossover study. AChEI = Acetylcholinesterase inhibitors. Rx = Dispensed Prescription.

***Treatment Groups.*** We inferred patient AChEI use by reconstructing courses of AChEI therapy from pharmacy claims data. To achieve a greater homogeneity among users’ disease stage and risk of adverse reactions ^8^, we restricted the AChEIs cohort to the first incident course of AChEI therapy, which was defined as their first course with at least a 180-day drug-free period. To ensure that patients were receiving medical care during the 180-day drug-free period and were not receiving the drug elsewhere, recipients’ had to be enrolled and to have at least one medical claim during the 180-day drug-free (baseline) period. We defined a course of AChEI therapy as beginning on the week the drug was first dispensed and ending on day 60 after a continuous gap in the drug supply of ≥ 60 days ( Figure 1).

The untreated comparison group consisted of Medicaid recipients 50 years and older with a dementia-like diagnosis who did not receive AChEI therapy. We established a 180-day baseline period during which recipients were enrolled and had at least one medical claim. The index date for individuals in the untreated group began at the first dementia-related outpatient visit that allowed for a 180-day baseline period. Starting time zero with a dementia-related outpatient visit established an indicated population that was engaging the health care system.

***Outcomes.*** As noted earlier, our primary clinical outcomes were gastrointestinal, psychological, respiratory, hematological and hepatic conditions, and death. We identified health care visits related to each clinical outcome in professional and facility claims using *Healthcare Cost and Utilization Project* (HCUP) *Clinical Classification Software* (CCS) codes (documented in Table 1). As a primary diagnosis typically indicates the reason for seeking medical care or the most important problem at the visit, we limited the outcome detection to the primary diagnosis codes. We tailored outcome classifications for each study design (described under Event Detection). Our analysis also measured the association of AChEI use with death.

***Potential confounding.*** We assessed demographic variables, comorbidities, drug therapy, and indicators of health care utilization as potential confounders. Comorbidity indices included HCUP comorbidity software version 3.2 and the modified RxRisk-V (RxRisk-Vm) score, which infers comorbidity using pharmacy claims ^9^. We measured health care utilization by considering the number of outpatient visits, hospitalizations, and emergency department (ED) visits, and we also accounted for use of hospice services and nursing home care.

We considered specific classes of medications as potential confounders—specifically, antianxiolytics, anticonvulsants, Parkinson’s treatment, antidepressants, antipsychotics, steroids, narcotics, respiratory agents, anticoagulants, corticosteroids, and sedatives. We treated the use of statin drugs as an indicator of health status because they are preferentially prescribed to healthier, less frail patients who are not at the end of life ^10^.

***Person time unit.*** We constructed the final analytic table using 1-week discrete time intervals; i.e., changes in covariate status, medication use and outcomes are captured weekly. This interval maximizes efficiency without omitting clinically important changes in patient outcome and covariate status. All database manipulation was conducted in SAS 9.2.

### Event detection

***Cohort design.*** We used an open cohort design with propensity score matching to explore associations between data on drug utilization and possible ADRs. We used propensity scores to address covariate imbalance using logistic regression models to predict AChEI treatment. We included confounders and risk factors in the propensity score models ^11^. Because we included risk factors along with confounders, we built separate propensity score models and matched cohorts for each study outcome. Two physicians who routinely treat patients with dementia independently selected variables to construct propensity score models. They discussed disagreements to arrive at consensus. Variables for each model are listed in Table 3.

Our analyses included propensity score matching followed by additional matching on key prognostic covariates ^12^. For example, we performed propensity matching with covariate matching whether an individual had a gastrointestinal visit during the baseline period when evaluating the gastrointestinal outcome. Analysis of death consisted of propensity score matching and covariate matching for baseline age and hospice care.

Clinical endpoints were intended to measure increased health care utilization associated with specific diagnoses. We defined episodes of care to differentiate clusters of events and to reduce the impact of immediate clinical exuberance associated with a new episode of care. A 4-week gap in claims for each clinical outcome was required to initiate a new episode. For each study endpoint, we calculated the incidence densities per 100 patient-years.

We established matched untreated cohorts using Mahalanobis metric matching ^13^. Baseline characteristics of patients in the AChEI-treated and matched untreated cohorts were compared using Student’s t-tests and chi-square tests. We used conditional multivariable Cox-proportional hazard models that allowed for recurrent events to assess the effect of AChEI on specific clinical endpoints ^14^. All statistical analyses were performed with Stata MP 9.2 for Windows.

***Case-crossover design.*** We established three 6-week time-windows (pre-treatment, first treatment, second treatment window) to assess acute and transient effects of AChEI treatment ( Figure 1). The index week for the pre-treatment window was the week following the most recent clinic visit for any condition during the baseline period.

**Figure 2.**
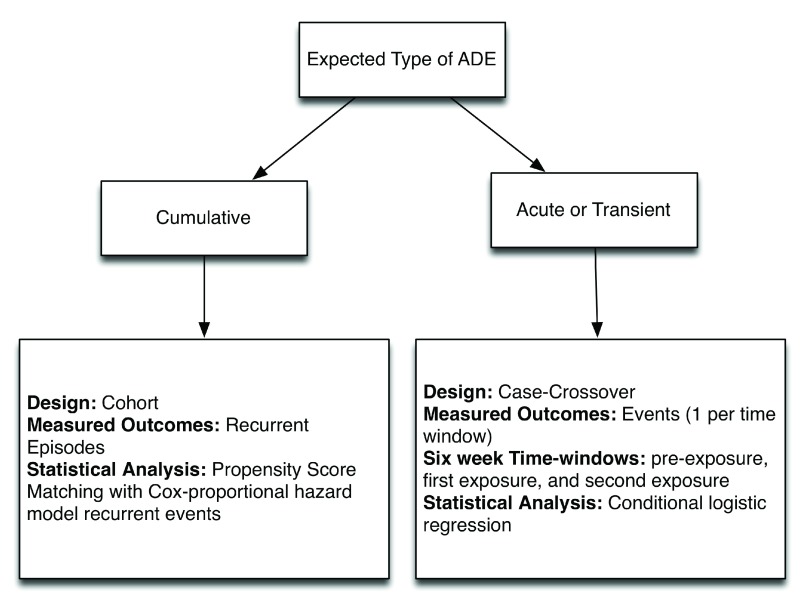
Study designs used for adverse event discovery and their purpose.

To capture ***acute*** effects of AChEI treatment, we used the week the AChEI was first dispensed as the index week for the first treatment window. We compared the odds of experiencing an event during that window with the odds of experiencing an event during the pre-treatment window to identify acute treatment effects. To evaluate the ***transience*** or ***stability*** of possible ADRs, we compared the odds of experiencing an event during the second treatment window to the odds of experiencing an event during the pre-treatment window. Patients were noted as having an event if they had a medical claim with the primary clinical diagnosis code of interest; we used only one event per time-window. Odds ratios between the referent and treatment windows were computed using conditional logistic regression. See Figure 2 for a summary of the two designs.

## Results

### Description of study population

**Figure 3.**
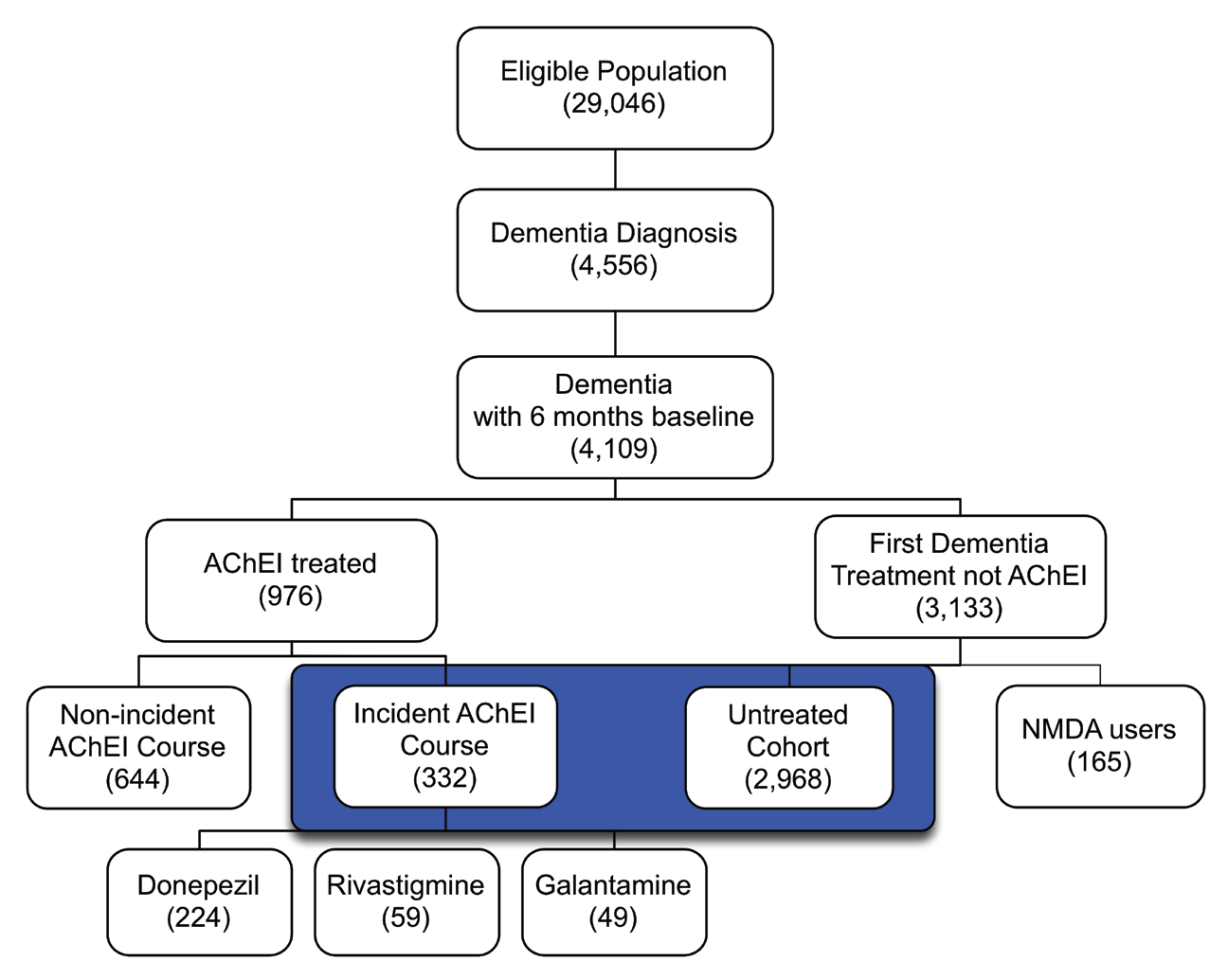
Dementia diagnosis and AChEI drug treatment in the eligible study population. Note: Groups highlighted in blue met the inclusion criteria for this study and were the primary comparison.

Of the 29,046 eligible patients in the study populations, 4,109 had a medical claim with a dementia diagnosis between 1/01/2003 and 12/31/2005. The AChEI-treated cohort consisted of 976 total users and 332 users with incident courses; of the latter, 224 were started on donepezil, 59 on rivastigmine, and 49 on galantamine. Because the numbers of incident users of specific AChEIs were small, we did not assess potential ADRs for individual drugs. In the AChEI-treated group the median duration of incident courses was 33.4 weeks with an interquartile range (IQR) from 15 to 68.5 weeks. The median proportion of weeks for which the AChEI-treated group was estimated to have access to the medication at least 1 day during the week was 100%, with an IQR of 95%–100%. The untreated cohort consisted of 2,968 patients who were diagnosed with dementia but did not receive medication to treat the disorder during the study period ( Figure 3).

Basic characteristics of the study population during the 6-month baseline period are presented in Table 2. Compared with the untreated population, incident AChEI users were slightly younger, had fewer HCUP comorbidities, fewer clinic visits, and a lower frequency of hospice care. Incident AChEI users also had a higher frequency of statin use and nursing home care. RxRisk-Vm scores and the average numbers of hospitalizations and ED visits were similar for AChEI users and non-users (untreated patients).

**Table 2.** Basic characteristics of the study population, 2003–2005.

Patient characteristics	AChEI cohort	Untreated cohort	P-value
	(n = 332)	(n = 2,968)	
Average age, years (SD)	76.4(11.4)	77.9(12.4)	0.02
Frequency female (Yes = 1)	71%	72.6%	0.53
Average number of HCUP comorbidities (SD)	1.1(1.5)	1.3(1.6)	<0.00
Average RxRisk-Vm (SD)	4.4(3.1)	4.5(3.3)	0.58
Average number of hospitalizations (SD)	0.19(0.45)	0.17(0.5)	0.44
Average number of ED visits (SD)	0.11(0.51)	0.11(0.58)	0.17
Average number of clinic visits (SD)	15.9(14.3)	16.7(16)	<0.00
Receiving hospice are (Yes = 1)	0.6%	3.2%	<0.00
Frequency of statin dispensed (Yes = 1)	25.6%	16.4%	<0.00
Frequency of nursing home stay (Yes = 1)	25.9%	14.4%	<0.00

AChEI = Acetylcholinesterase inhibitors HCUP = Healthcare Cost and Utilization project SD = Standard deviation % = Percent ED = Emergency department RxRisk-Vm = modified RxRisk-V

After propensity score matching for each clinical endpoint, the two groups were similar on all variables for each outcome-based cohort, except the average number of ED visits, which was slightly higher in the untreated matched groups for the evaluation of respiratory and hepatic episodes ( Table 3). In general, the lack of statistically significant differences between the AChEI-treated and untreated groups on propensity-adjusted variables suggests a balance in measured covariates between treatment groups.

**Table 3.** Baseline comparisons for all variables included in each matched cohort analysis.

Baseline	AChEI	Gastrointestinal		Psychological		Respiratory		Hepatic		Hematologic		Death	
	Tx	UnTx	P-value	UnTx	P-value	UnTx	P-value	UnTx	P-value	UnTx	P-value	UnTx	P-value
Age (yrs)	76.4	78.2	0.39	76.5	0.89	76	0.66	74.7	0.06	74.9	0.1	76.5	0.29
No. HCUP comorbidities	1.11	1.09	0.43	1.06	0.96	1.04	0.57	0.93	0.06	1.02	0.63	1	0.39
RxRisk Score	4.38	4.5	0.95	4.57	0.30	4.63	0.44	4.7	0.2	4.2	0.39	4.33	0.70
Sex (Male)	89	74	0.17	82	0.52	75	0.20	85	0.72	96	0.53	74	0.17
Statin Med	85	94	0.38	89	0.66	81	0.72	85	0.2	82	0.76	85	1
Hospice care	2	2	1	1	1	2	1	3	1	5	0.25	2	1
No. ED visits	0.11	0.18	0.80	1.12	0.7	0.17	0.04*	0.17	0.04*	0.17	0.2	0.12	0.39
No. Hospitalizations	0.19	0.16	0.11	0.23	0.33	0.15	2.0	0.13	0.06	0.21	0.15	0.18	0.59
Clinic visits (>5)	284	295	0.16	285	1	280	0.61	284	1	277	0.37	282	0.80
No. with GI episode	65	65	1									
No. with hematologic episode	27									27	1	
No. with hepatic episode	15							15	1			
No. with psychological episode	84			84	1							
No. with respiratory episode						45	1					
Respiratory meds	6					11	0.27					
Steroids	34	43	0.23	45	0.18	39	0.53			47	0.11	
NSAIDs	124	137	0.29							113	0.35	
Gastroprotective meds	117	117	1									
Anxiolytics	103	103	1	98	0.67	111	0.49	88	0.19			109	0.60
Anticonvulsants	85			81	0.71	90	0.66	98	0.25	97	0.29	77	0.43
Parkinson’s Meds	37			26	0.19							40	0.81
Antipsychotics	135	135	1	128	0.55	111	0.05	140	0.67			149	0.27
Antidepressants	209	222	0.27	196	0.26	211	0.86	200	0.46			203	0.63
Narcotics	157	165	0.53	157	1	159	0.87	161	0.76			165	0.53
Sedatives	54	55	0.91	65	0.26	57	0.75	45	0.32			47	0.43
Liver toxic meds	80							78	0.77			
Diag. alcohol abuse	9	11	0.82	6	0.61	11	0.65	6	0.58	13	0.52	9	1
Diag. deficiency anemia	39	34	0.63					44	0.65	40	0.87	28	0.22
Diag. blood loss anemia	4	3	1					4	1	2	0.69	1	0.38
Diag. pulmonary disease	57					62	0.38			67	0.30	47	0.35
Diag. depression	65	69	0.70	71	0.49							58	0.50
Diag. diabetes	71	70	0.92	77	0.05					68	0.76	66	0.63
Diag. hypertension, complicated	150											139	0.36
Diag. hypothyroidism	62	69	0.49	67	0.60	66	0.67			56	0.53	58	0.67
Diag. liver disease	9	6	0.61			9	1	8	1	7	0.80	6	0.61
Diag. fluid and electrolyte disorder	47	40	0.36			48	0.91	33	0.1	56	0.33	43	0.63
Diag. paralysis	7											8	1
Diag. peripheral vascular disorder	72	77	0.65			70	0.85	60	0.24			64	0.42
Diag. metastatic cancer	1	2	1	1	1					5	0.22	0	1
Diag. psychoses	84	79	0.63	75	0.30							88	0.70
Diag. pulmonary circulation disorder	5					4	1	3	0.73	6	1	4	1
Diag. obesity	7	11	0.34			7	1	11	0.46			9	0.80
Diag. renal failure	3									2	1	2	1
Diag. chronic peptic ulcer disease	2	2	1	1	1					1	1	3	1
Diag. coagulation deficiency	12	11	1					6	0.18	13	1	13	1
Diag. valvular disease	18					19	1			16	0.86	19	1
Diag. weight loss	38	33	0.50	40	0.80	39	0.90			40	0.80	35	0.80

p-value <0.05 No. = number

Table 4 presents the incidence densities per 100-person years and 95% confidence intervals for the complete untreated population and propensity-matched comparisons. Table 5 presents the hazard rates for all unadjusted and matched comparisons.

**Table 4.** Crude incidence densities (per 100-person years) of target events in AChEI treated and untreated groups.

	AChEI treated cohort			Untreated cohort			Matched untreated cohort		
	N	Incidence Density	95% CI	N	Incidence Density	95% CI	N	Incidence Density	95% CI
**Expected reactions**								
Gastrointestinal episodes	78	27.6	24.5, 30.7	878	25.7	24.8, 26.5	63	17.3	15.1, 19.5
Psychological episodes	141	50	45.8, 54.2	1399	40.9	39.8, 42.0	159	56.4	52.5, 60.3
**Suspected reactions**								
Respiratory episodes	91	32.2	28.9, 35.6	1004	29.4	28.4, 30.3	84	23.8	21.2, 26.4
**Unexpected reactions**								
Hematological episodes	70	24.8	21.8, 27.8	651	19.0	18.3, 19.8	55	14.9	12.9, 16.9
Hepatic episodes	13	4.6	3.3, 5.9	121	3.5	3.2, 3.9	12	3.2	2.3, 4.1
**Death**	83	21.1	18.8, 23.4	1100	32.2	31.2, 33.1	92	23.6	21.1, 26.1

**Table 5.** Unadjusted and matched analysis comparing target outcomes for groups treated and not treated to AChEI therapy.

Outcome	Crude			Propensity matched		
	HR	*P* **-value**	95% CI	HR	*P* **-value**	95% CI
**Expected reactions**					
Gastrointestinal episodes	1.01	0.95	0.8, 1.27	2.02	<0.00	1.28, 3.2
Psychological episodes	1.12	0.2	0.94, 1.33	1.13	0.35	0.87, 1.47
**Suspected reactions**					
Respiratory episodes	1.03	0.76	0.83, 1.28	1.21	0.35	0.81, 1.79
**Unexpected reactions**					
Hematological episodes	1.26	0.07	0.98, 1.62	2.32	0.00	1.47, 3.67
Hepatic episodes	1.18	0.56	0.67, 2.1	1.77	0.24	0.68, 4.6
**Death**	0.66	<0.01	0.52, 0.82	1.07	0.5	0.74, 1.54

***Crude analyses.*** In bivariate analysis ( Table 5) we did not observe a higher rate of gastrointestinal episodes in the group treated with AChEIs compared to the untreated group. The rates of psychological episodes, respiratory episodes, hematological episodes, and hepatic episodes were slightly higher, but not statistically significantly, in the group treated with AChEIs compared to the untreated group. The rate of death in the group treated with AChEIs was significantly lower than in the untreated group.

***Propensity-matched analyses.*** We observed significantly higher rates of gastrointestinal episodes (Hazard Ratio [HR]: 2.02; 95% CI: 1.28 - 3.2) and hematologic episodes (HR: 2.32; 95% CI: 1.47 - 3.67) in the AChEI-treated group than in the propensity-matched untreated group ( Table 5). For psychological episodes, respiratory episodes, and hepatic episodes, we observed higher, but not statistically significant, rates in the AChEI-treated group than in the propensity-matched untreated group. We observed a weak and non-significant association between AChEI treatment and mortality.

***Case-crossover analysis.*** In crossover analysis we did not observe increased odds of experiencing gastrointestinal events during either the first or second treatment windows. We observed an acute, but non-significant, effect of AChEI treatment on the odds of experiencing a psychological event during the first-treatment window; this was not sustained during the second-treatment window. We observed acute, but non-significant, effects of AChEI treatment on the odds of experiencing respiratory event and hematological events during the first-treatment window; both rates appeared to decrease during the second-treatment window. The acute effect of AChEI treatment on the odds of experiencing a hepatic event during the first-treatment window was imprecise and appeared to decrease during the second-treatment window ( Table 6).

**Table 6.** Cohort crossover design: evaluation of acute and transient effects of AChEI treatment.

Type of reactions	Measures	Pre-treatment	1 ^st^ treatment window	2 ^nd^ treatment window
		(n = 271)	(n = 312)	(n = 303)
**Expected reactions**			
Gastrointestinal events	Events	11	10	11
	OR (95% CI)	†	0.7 (0.27, 1.84)	0.86 (0.29, 2.6)
	p-value		0.47	0.78
Psychological events	Events	28	39	30
	OR (95% CI)	†	1.5 (0.72, 3.3)	0.86 (0.40, 1.9)
	p-value		0.26	0.7
**Suspected reactions**			
Respiratory events	Events	12	14	15
	OR (95% CI)	†	1.4 (0.44, 4.4)	1.2 (0.37, 3.9)
	p-value		0.57	0.76
**Unexpected reactions**			
Hematological events	Events	5	13	9
	OR (95% CI)	†	3 (0.97, 9.3)	1.75 (0.51, 6.0)
	p-value		0.06	0.37
Hepatic events	Events	2	6	1
	OR (95% CI)	†	5 (0.58, 42.8)	0.5 (0.05, 5.5)
	p-value		0.14	0.57

## Discussion

We developed a cohort-based framework for using population-based administrative data to identify known ADRs and to discover ADRs that may have gone unnoticed during clinical trials. We evaluated AChEI therapy in people with dementia, considering a composite of possible ADRs─ i.e., expected, suspected, unexpected reactions, and death ─to demonstrate that our analytic techniques produced expected results. We used propensity score matching and a within-subject design in an attempt to handle confounding. Our pilot study examined data from patients diagnosed with dementia for both cumulative effects of AChEI treatment and acute effects following initiation of AChEI therapy. We demonstrated this approach with Medicaid data from the state of Utah; nonetheless, the framework presented here can be transferred for use with other health insurer databases, including the Medicare Parts A, B, and D data now available.

A pervasive issue in pharmacoepidemiologic studies is confounding by indication ^15^. This problem arises because factors that influence treatment choices made by clinicians also influence outcomes. Confounding by indication can bias the crude association between drug treatment and outcomes in either direction and with unknown magnitude. Propensity score models are one method used in pharmacoepidemiologic studies to balance measured confounders with the goal of making the treatment groups exchangeable.

In this study, we addressed confounding by indication by developing propensity score models for each study outcome. Theoretical confounders available in the data were included in each model to reduce bias. Before matching, the untreated group appeared to be frailer than the treated group; they had a higher proportion of hospice care, more comorbidity, and a lower proportion of statin users, which suggested less aggressive care because of poorer health. As one would expect, the unadjusted analysis made AChEI treatment appear protective against mortality when compared with the untreated group (HR: 0.66; 95% CI: 0.52 - 0.82), which is not supported by clinical trials or other observation studies ^16, 17^. After propensity and covariate matching we found no difference between the AChEI-treated and untreated groups (HR: 1.07; 95% CI: 0.74 - 1.54). This illustrates the importance of addressing confounding by indication when designing ADR surveillance systems.

An alternative approach to addressing confounding is to use inverse probability weighting (IPW) methods to model time-varying treatments and confounders. In simulation studies, these methods were less biased than conventional methods when time-varying confounding was present ^18^. When allowing treatment to be time-varying, we observed gastrointestinal disturbance and discovered hematological disturbance; we noted the same findings as if follow-up began at initiation of drug treatment (data not shown). Future work should explore the presence of time-varying confounding and the benefits of using IPW methods to discover novel ADRs associated with drug treatments.

To evaluate possible acute and transient effects of AChEI treatments, we employed a type of case-crossover analyses. Typically in case-crossover analyses, events are compared between event and control time-windows for each individual. A major benefit of this within-subject design is that each person acts as his or her own control ^19, 20^. It also accounts for confounding by indication and other time-invariant and difficult-to-measure confounders. The drawback of such designs involves changes in treatment utilization that are influenced by health status or the study endpoints in question ^21^. For example, when day-level drug utilization data are inferred from dispensing history, determining whether adverse effects are truly transient or the result of a decrease or discontinuation of drug treatment is difficult. Ultimately, we deemed the within subject analysis to be an excellent complement to the propensity score approach because of its ability to discover acute and transient effects and for its simplicity and ability to remove time-invariant confounding by indication.

## Hypothesis generating and confirmation

The framework described here provides a structured approach for confirming expectations by evaluating known ADRs and discovering new ADR safety signals, such as the association we found between AChEI use and hematological disturbance. In support of the analytical effectiveness of these procedures, our approach confirmed an association with an expected reaction, gastrointestinal disturbances. The findings from the two study designs, however, were not consistent. Our inability to find an acute increase of gastrointestinal events in the first-treatment time window may be attributable to insensitivity of claims-based coding to identify symptoms of gastrointestinal disturbance.

Despite the fact that our approach detected a significant association with one expected reaction, gastrointestinal disturbance, it failed to identify a strong positive association with the second expected reaction, psychological disturbance. We did show a higher rate of psychological episodes in the propensity-matched analysis; nevertheless, the association was not statistically significant. We did; nonetheless, observe higher odds of experiencing psychological events in the first-treatment time window than in the pretreatment time window using the within subject design. Even though the higher odds was expected, it was not statistically significance. This result can likely be attributed to a combination of factors. First, is the low power in the within subject design and second may be insensitivity of claims-based coding to identify symptoms of psychological disturbance.

We discovered no clear associations between AChEIs and respiratory disturbance or death. In a recent sequence symmetry analysis, the initiators of AChEI had no detectable increased rate of complications of chronic airway disorders ^25^. We found no clear evidence of an increase or decrease in mortality associated with AChEI treatment in published studies or meta-analysis to which to compare our results ^16^.

Our analysis of unexpected reactions discovered a statistically significant positive association between AChEI treatment and hemotological episodes. Hematological events also appeared to be positively associated with early AChEI treatment. A detailed review of results with hematological event subcategories (not reported here) found that the rate of anemia was much higher in the AChEI-treated group than in the untreated group during the first 6 weeks of drug treatment. Further analysis is required to determine if this higher rate is causally associated with initiating anti-dementia drug treatment. At present, no known pharmacologic or empirical reasons can explain how AChEI drugs cause hematological toxicity.

The incidence of hepatic disturbance appeared to be higher in the treated group, although non-significant, in both the within subject and propensity matched design. Hepatotoxicity was a major safety concern with tacrine, which is the reason why it is no longer a commonly used; hepatotoxicity has not been reported for other AChEIs ^26^. Larger observational studies are needed to determine whether an association between AChEIs and hepatotoxicity exists.

## Limitations

The results from this study are considered hypothesis generating rather than identifying causal treatment effects. Causal studies require validation of treatments, outcomes, and covariate classifications. Furthermore, causal studies require a stronger theoretical understanding and explication of the underlying causal relationships between the treatment and outcomes.

We compared AChEI-treated patients with an incident AChEI course of therapy, to an untreated cohort of patients with a dementia diagnosis. Other options were to compare directly the safety of AChEI products with one another or to compare the safety of AChEI therapy with the safety of other classes of medications used to treat such patients’ dementia. We were not able to compare individual drug products. Treatment with AChEIs is not directly comparable to treatment with memantine, a glutamaterginc N-methyl D-aspartate (NMDA) receptor antagonist, because memantine is typically not the first-line treatment for dementia; rather it is used in addition to an AChEI therapy, complicating any comparison.

In pharmacoepidemiologic studies, an untreated referent group can also be defined as patients with an incident course of a medication that is not associated with the indication or evaluated outcomes. This type of “active control group” is likely to be more similar to the treated group in regard to the activation of the health care system than the indicated but untreated group ^17^. Drug dispensing indicates that the patient has activated the health system. In addition, prescription of a new medication is likely to result in closer monitoring and evaluation of an individual’s health status. The primary concern when comparing treated with untreated groups is under-recording of health conditions, making the members of the comparison group seem healthier than they really are, which can lead to overestimation of the effects of drug treatment.

Because of the multiple outcomes in this study, we were unable to identify a single medication that could yield comparable cohorts for all events. Instead, we used a dementia-related visit, not drug dispensing, as the index date for the untreated group. For both cohorts, the median amount of time to a clinic visit following the index date was 3 weeks, and the longitudinal visit process was also similar. These patterns suggest that health care access and followup may have been similar for the two groups.

Another limitation of this study is the small number of subject in the AChEI treatment group. This markedly limited our ability to confirm the expected adverse effects of AChEI treatment and discover adverse events that may have gone undetected in clinical trials.

## Future research

The discovery of an association between a drug treatment and a theoretical reaction, an idiosyncratic reaction, or death is considered hypothesis generating or signal detection. Confirmation requires additional observational and possibly experimental studies. Ideally, discovered associations would first be confirmed or further characterized in large, disparate data sources to reproduce evidence of the association across different populations. In May 2008, the FDA published The Sentinel Initiative report to present the national strategy for monitoring medical product safety ^27^. Their approach primarily establishes a nationwide health information network for confirmation of safety signals across multiple large databases. Additional observational studies along with richer clinical information such as electronic health records or prospectively designed studies, however, may be needed to characterize the *causal* relationship between a drug treatment and the adverse outcome.

## Data availability

The raw data are available upon request. IRB approval and signed Data Use Agreements with the Utah Department of Health. For more information, please contact the corresponding author Brian Sauer.

## Ethical considerations

The primary ethical consideration is the privacy and confidentiality of patient data. Limited datasets were used that restricted the use of direct patient identifiers. Data are stored on secured servers and only shared according to IRB policy and state data use agreements.
