# Supplementary material for: Methodological framework to identify possible adverse drug reactions using population-based administrative data
Source: F1000Res. 2014 Oct 29;3:258. [Version 1] doi: 10.12688/f1000research.4816.1 (PMC4490782; doi:10.12688/f1000research.4816.1)
Supplement: Peer review history

# Peer review: Referee response for version 1

**DOI:** 10.5256/f1000research.5140.r9448

**Published:** 2015-07-10

**Authors:** Alison Bourke

**Affiliations:** 1 Cegedim Strategic Data, London, UK

This is an interesting paper, and as stated, it would be useful to see the methodology repeated on larger datasets to increase the power. One small point - the authors say that their inability to find an acute increase of gastrointestinal events in the first-treatment time window may be attributable to insensitivity of claims-based coding to identify symptoms of gastrointestinal disturbance. Could this also be because they were only looking for the outcome as a primary diagnosis, and GI disturbance may be consider less important? Would it have been possible to use prescribing of certain therapeutic agents as a proxy of GI events

I have read this submission. I believe that I have an appropriate level of expertise to confirm that it is of an acceptable scientific standard.

# Peer review: Referee response for version 1

**DOI:** 10.5256/f1000research.5140.r9151

**Published:** 2015-07-01

**Authors:** Yajaira M. Bastardo

**Affiliations:** 1 Faculty of Pharmacy, Central University of Venezuela, Caracas, Venezuela

Overall Comments: This study presents a framework for discovering ADRs using population based administrative data sources. The approach is exemplified by use of AChEIs treatment on dementia. Overall, the study is very well done. However, there is an issue that needs to be clarified as following:

On page 4, paragraph 3, the authors state that they treated the use of statin drugs as indicators of health status because they are preferably prescribed to less frail who are not at the end of life. Even though the statement seems reasonable the reference they provided does not support it.

The results of these pilot study shows that use the framework for discovering ADRs using population based administrative data a source is promising as a hypothesis generating approach.

I have read this submission. I believe that I have an appropriate level of expertise to confirm that it is of an acceptable scientific standard.

# Peer review: Referee response for version 1

**DOI:** 10.5256/f1000research.5140.r6950

**Published:** 2015-02-04

**Authors:** Qayyim Said

**Affiliations:** 1 Division of Pharmaceutical Evaluation and Policy, University of Arkansas for Medical Sciences, Little Rock, AR, USA

Overall Comments: This study explored methods for evaluating ADRs in claims data using an example of effects of AChEIs treatment on Dementia patients. Overall, the study is very well done. However, there are a few issues that need to be clarified as following:

1. While identifying patients with first incident course of AChEI therapy, did the authors apply any criteria for dosage, strength, or duration of the incident treatment?
2. What was the index date for the treated group? The authors explicitly refer to the index date for the untreated group, but not for the treated group.
3. Why did the authors choose "death" as one of the outcomes? Was it based on any prior evidence in the case of AChEI therapy?
4. On page 5, in the last paragraph of left panel, I am not sure what exactly the authors are trying to say in the following sentence: "Clinical endpoints were intended to measure increased health care utilization associated with specific diagnoses." It may be helpful to clarify it.
5. The authors have done well to balance measured confounding by using propensity score methods. However, there may also unmeasured (unobserved) confounding in clinical settings that may have implications for some of the results (e.g. the inability to find some of the ADRs). In future studies, methods to control for unobserved confounding (e.g. instrumental variables) may also be used.
6. While discussing (page 11) reasons for inconsistencies in the results from the two study designs, the authors speculate that their "inability to find an acute increase of gastrointestinal events in the first-treatment time window may be attributable to insensitivity of claims-based coding to identify symptoms of gastrointestinal disturbance." The authors attribute similar reason for failing to find evidence of psychological disturbances. It would be helpful if the authors could provide an example of insensitivity of claims-based coding to identify symptoms in a disease state.

I have read this submission. I believe that I have an appropriate level of expertise to confirm that it is of an acceptable scientific standard.
